# The Congenital Heart Disease Genetic Network Study: Cohort description

**DOI:** 10.1371/journal.pone.0191319

**Published:** 2018-01-19

**Authors:** Thanh T. Hoang, Elizabeth Goldmuntz, Amy E. Roberts, Wendy K. Chung, Jennie K. Kline, John E. Deanfield, Alessandro Giardini, Adolfo Aleman, Bruce D. Gelb, Meghan Mac Neal, George A. Porter, Richard Kim, Martina Brueckner, Richard P. Lifton, Sharon Edman, Stacy Woyciechowski, Laura E. Mitchell, A. J. Agopian

**Affiliations:** 1 Department of Epidemiology, Human Genetics and Environmental Sciences, UTHealth School of Public Health, Houston, Texas, United States of America; 2 Department of Pediatrics, University of Pennsylvania Perelman School of Medicine, Philadelphia, Pennsylvania, United States of America; 3 Department of Cardiology, Children’s Hospital Boston, Boston, Massachusetts, United States of America; 4 Department of Pediatrics, Columbia University Medical Center, New York, New York, United States of America; 5 Department of Medicine, Columbia University Medical Center, New York, New York, United States of America; 6 Department of Epidemiology, Mailman School of Public Health, Columbia University, New York, New York, United States of America; 7 Cardiothoracic Unit, Great Ormond Street Hospital, London, England; 8 University College London Institute of Cardiovascular Science, University College London, London, England; 9 Mindich Child Health and Development Institute, Icahn School of Medicine at Mount Sinai, New York, New York, United States of America; 10 Department of Pediatrics, Icahn School of Medicine at Mount Sinai, New York, New York, United States of America; 11 Department of Genetics and Genomic Sciences, Icahn School of Medicine at Mount Sinai, New York, New York, United States of America; 12 Department of Pediatrics, University of Rochester Medical Center, Rochester, New York, United States of America; 13 Section of Cardiothoracic Surgery, University of Southern California Keck School of Medicine, Los Angeles, California, United States of America; 14 Department of Genetics, Yale University School of Medicine, New Haven, Connecticut, United States of America; 15 Yale Center for Mendelian Genomics, Yale University School of Medicine, New Haven, Connecticut, United States of America; 16 Department of Internal Medicine, Yale University School of Medicine, New Haven, Connecticut, United States of America; 17 Howard Hughes Medical Institute, Yale University School of Medicine, New Haven, Connecticut, United States of America; Northwestern University, UNITED STATES

## Abstract

The Pediatric Cardiac Genomics Consortium (PCGC) designed the Congenital Heart Disease Genetic Network Study to provide phenotype and genotype data for a large congenital heart defects (CHDs) cohort. This article describes the PCGC cohort, overall and by major types of CHDs (e.g., conotruncal defects) and subtypes of conotrucal heart defects (e.g., tetralogy of Fallot) and left ventricular outflow tract obstructions (e.g., hypoplastic left heart syndrome). Cases with CHDs were recruited through ten sites, 2010–2014. Information on cases (N = 9,727) and their parents was collected through interviews and medical record abstraction. Four case characteristics, eleven parental characteristics, and thirteen parent-reported neurodevelopment outcomes were summarized using counts and frequencies and compared across CHD types and subtypes. Eleven percent of cases had a genetic diagnosis. Among cases without a genetic diagnosis, the majority had conotruncal heart defects (40%) or left ventricular outflow tract obstruction (21%). Across CHD types, there were significant differences (p<0.05) in the distribution of all four case characteristics (e.g., sex), four parental characteristics (e.g., maternal pregestational diabetes), and five neurodevelopmental outcomes (e.g., learning disabilities). Several characteristics (e.g., sex) were also significantly different across CHD subtypes. The PCGC cohort is one of the largest CHD cohorts available for the study of genetic determinants of risk and outcomes. The majority of cases do not have a genetic diagnosis. This description of the PCGC cohort, including differences across CHD types and subtypes, provides a reference work for investigators who are interested in collaborating with or using publically available resources from the PCGC.

## Introduction

Congenital heart defects (CHDs) occur in approximately 1% of births and are among the most common and serious birth defects [1, 2]. While advances in treatment have reduced CHD-related mortality, CHDs remain the leading cause of birth defect-related infant deaths [3]. Moreover, the growing numbers of CHD survivors are at risk for a range of disease-related morbidities [4, 5] and have reduced life-expectancies compared to their unaffected contemporaries [4].

CHDs include a broad spectrum of malformations that differ with respect to morphology, physiology, and clinical outcome. Although CHD risk is thought to be influenced by both environmental and genetic factors, relatively few specific CHD risk factors have been identified and the extent to which the etiology of different CHDs differ or overlap is unknown. Large epidemiological studies, such as the National Birth Defect Prevention Study, have identified a few non-genetic risk factors for CHDs including maternal pre-gestational diabetes, obesity, and smoking [6–10]. To accelerate understanding of the genetic contribution to CHDs, the National Heart, Lung, and Blood Institute formed the Pediatric Cardiac Genomics Consortium (PCGC). The PCGC designed and implemented the Congenital Heart Disease GEnetic NEtwork Study (CHD GENES) to establish the resources required to undertake comprehensive studies of the genetics of CHDs.

The rationale for, design of, and early results from CHD GENES have been described [11–14]. In addition, genotype array, exome sequence, whole genome sequence, and RNA sequence data from CHD GENES participants have been and will continue to be posted to dbGAP (dbGAP Accession: phs000571.v3.p2, January 2016). In this article, we provide a description of the phenotypes, characteristics, and selected parent-reported neurodevelopmental outcomes of the PCGC cohort, as a resource for the broader CHD research community.

## Methods

### Study population

Subject recruitment and data collection for CHD GENES have been described [11]. Briefly, subjects were recruited from five main sites (Children’s Hospital of Philadelphia, Columbia University Medical Center, Harvard Medical School including Boston Children’s Hospital and Brigham and Women’s Hospital, Icahn School of Medicine at Mount Sinai, and Yale School of Medicine) and four satellite sites (Children’s Hospital of Los Angeles, Cohen Children’s Medical Center, University College London, and University of Rochester Medical Center) from December 2010 through November 2014. Recruitment methods were center-specific, but generally included ascertainment of cases at the time of hospital admission or an outpatient visit. The study protocol was approved by an Institutional Review Board for each site. All study participants (or their parent/guardian) provided written informed consent. The Institutional Review Board at the University of Texas Health Science Center at Houston approved the study protocol for the data analyzed and presented in this article.

Patients with any diagnosis of CHD (except as noted below), regardless of sex, age, and race/ethnicity were eligible to participate. Patients with a genetic diagnosis were eligible to participate, but preference for enrolling such patients may have varied across study sites. Patients with isolated patent foramen ovale, prematurity-related isolated patent ductus arteriosus, pulmonary stenosis related to a twin-twin transfusion, and cardiomyopathy without a CHD were not eligible. Cardiac diagnoses were confirmed by review of imaging (e.g., echocardiogram) and operative reports. Information on genetic testing, genetic physical exams, and extracardiac malformations was abstracted from medical records. In addition, information on cases and their parents was obtained during subject and family interviews. Cases that did not participate in the interviews were excluded from this report.

Data collected by interview included race/ethnicity, sex, birth weight, and maternal and paternal ages at the time of the cases’ birth. Data were also collected on maternal characteristics, including pre-pregnancy height and weight (to calculate pre-pregnancy body mass index), pre-gestational diabetes, gestational diabetes, epilepsy or seizure during pregnancy, and education level. For cases who were ≤1 year of age at recruitment, interview data were also collected on maternal smoking and alcohol use during the first trimester, any folic acid supplementation six months before pregnancy, and parity. For cases who were >1 year at recruitment, the interview included questions related to neurodevelopmental outcomes (e.g., attention deficit hyperactivity disorder, autism spectrum).

CHD diagnoses assigned using the International Paediatric and Congenital Cardiac Codes (http://www.ipccc.net/) were manually reviewed by two of the authors (S.E and E.G.) and cases were assigned to one of seven types of CHDs: laterality disorder (LAT), conotruncal heart defect (CTD), atrioventricular septal defect (AVCD), left ventricular outflow tract obstruction (LVOT), right ventricular outflow tract obstruction (RVOT), atrial septal defect (ASD), and other. These groups are based on subsets of lesion that are thought to share genetic and mechanistic underpinnings and are defined in Table 1. Cases were categorized using a hierarchical approach. First, cases with a laterality disorder, regardless of other findings, were placed in LAT. Next, cases with abnormal conotruncal anatomy (including specific subtypes of isolated ventricular septal defects), regardless of associated left or right sided obstruction or atrioventricular canal anomalies, were placed in CTD. Then, cases with atrioventricular canal abnormalities with normally related great arteries were categorized as AVSD and cases with left or right sided obstructive lesions with normally related great arteries and normal atrioventricular canals were assigned to LVOT or RVOT, respectively. Finally, cases with an isolated secundum or sinus venosus type atrial septal defect were assigned to ASD. Cases with any other CHD diagnosis were assigned to the other group.

**Table 1:** Diagnostic types of congenital heart defect in the Pediatric Cardiac Genetic Consortium Cohort.

Diagnostic Type	Abbreviation	Description
Laterality Disorder	LAT	Includes cases with at least one of the following laterality disorders: dextrocardia, interrupted inferior vena cava, atrial situs abnormalities (i.e. ambiguous/inversus), L-transposition of the great arteries (ventricular inversion), asplenia/polysplenia, bronchial isomerism, abdominal laterality disorder, intestinal malrotation.
Conotruncal heart defect	CTD	Tetralogy of Fallot, truncus arteriosus, interrupted aortic arch, double outlet right ventricle, d-transposition of the great arteries, isolated conoventricular or posterior malalignment or conoseptal hypoplasia type ventricular septal defect, and isolated aortic arch anomalies
Atrioventricular canal defect	AVCD	Primum atrial septal defect, transitional atrioventricular canal defect, complete atrioventricular canal defect, isolated cleft mitral valve
Left ventricular outflow tract obstruction	LVOT	Bicuspid aortic valve/aortic valve stenosis, coarctation of the aorta, hypoplastic left heart syndrome^a^, mitral valve anomalies
Right ventricular outflow tract obstruction	RVOT	Triscupid valve atresia/stenosis with or without pulmonary valve atresia/stenosis with normally related great arteries, pulmonary valve atresia/stenosis with normally related great arteries
Atrial Septal Defect	ASD	Secundum or sinus venosus type
Other		Complex malformations (e.g., double inlet left ventricle), isolated venous or coronary artery anomalies, isolated muscular ventricular septal defect, other conditions

^a^ Does not include variants of hypoplastic left heart syndrome such as malaligned atrioventricular canal defect or double outlet right ventricle with mitral atresia.

Based on data from the interviews and medical records, cases were classified as either having 1) an identified genetic diagnosis (i.e. a syndrome or genetic alteration thought to explain the associated CHD), or 2) no genetic diagnosis. For simplicity, we refer to such cases as “syndromic” and “nonsyndromic”, respectively. Cases classified as nonsyndromic by this scheme may have had additional non-cardiac anomalies or reported neurodevelopmental deficits.

### Statistical analysis

For syndromic cases, we reported counts and frequencies for each specific diagnosis. Given the clinical heterogeneity within this group, we excluded syndromic cases from subsequent analyses. For nonsyndromic cases, parental characteristics, case characteristics, and parent-reported neurodevelopmental outcomes were described using counts and frequencies for discrete variables, and means and standard deviations or median and range for continuous variables. Due to differences in the education systems in the United States and United Kingdom, we excluded women who were educated in the United Kingdom in our description of maternal education. Further, we restricted our analyses of neurodevelopmental outcomes to cases who were ≥5 years of age at recruitment, since neurodevelopmental deficits may be under-diagnosed in younger children. In addition to assessing each of 13 parental-reported (yes/no) neurodevelopmental outcomes, we created a composite neurodevelopmental outcome variable, indicating a positive parental report for at least one of four conditions: developmental delay, learning disability, mental retardation, or autism spectrum disorder [13].

We used the chi-square test (or Fisher’s exact test when >20% of cells had an expected cell count <5) to compare the distribution of categorical variables across types of CHDs. For continuous variables, we used ANOVA or the Kruskal-Wallis test to compare the mean or median, respectively, across types of CHDs. For ANOVA analyses, we used Levene’s test to check the assumption of homogeneity of variance. If Levene’s test was significant (p<0.05), we used Welch’s ANOVA. Analyses of all variables, except neurodevelopmental outcomes, were repeated in the subset of cases who were ≤1 year of age at recruitment for the following reasons: 1) inaccurate recall of characteristics or events before or during pregnancy is of greater concern for cases ascertained at older ages than at younger ages; and 2) the distribution of characteristics across types of CHDs may be influenced by survival. Because of the heterogeneity within type of CHDs, analyses were also repeated to compare specific subtypes in the two largest types of CHDs—CTD and LVOT cases. These analyses were restricted to include subtypes that included at least 200 cases. For LVOT, cases with aortic stenosis were combined with cases with bicuspid aortic valve to create a subtype called ‘aortic valve disease.’

Because differences in the distribution of neurodevelopmental outcomes across types of CHDs may be influenced by factors other than the CHD diagnosis, we used logistic regression to control for potential confounders determined *a priori* from the literature [15]: maternal education, case race/ethnicity, sex, birth weight (low [<2,500g], normal [2,500–4,000g], high [>4,000g]), and extracardiac malformations (yes/no). Further, as neurodevelopmental deficits may be under-diagnosed in younger cases, we also adjusted for case age at the time of recruitment. Adjusted analyses were not conducted for the CTD and LVOT subtypes because of the relatively small numbers of cases with specific outcomes (e.g., double outlet right ventricle with autism spectrum, N = 4).

All analyses were conducted using SAS version 9.4 (SAS Institute Inc., Cary, NC). P-values <0.05 were considered statistically significant.

## Results

Data were available for 9,727 cases, including 1,034 (11%) with a genetic diagnosis. The most common syndromic diagnoses were trisomy 21 (38%) and DiGeorge syndrome (DGS)/Velocardiofacial syndrome (VCFS)/22q11.2 deletion (24%) (Table 2). Among cases with trisomy 21, the most common CHDs were AVCDs (52%) and CTDs (35%). Among cases with DGS/VCFS/22q11.2 deletion, the most common CHD was CTDs (96%) (S1 Table).

**Table 2:** Syndromic diagnoses in the Pediatric Cardiac Genetic Consortium Cohort.

Syndrome	N (n = 1,034)	%
Alagille	19	1.8
CHARGE	24	2.3
DGS/VCFS/22q11.2 Deletion	251	24.3
Ehlers Danlos syndrome	9	0.9
GATA4^a^	13	1.3
Goldenhar	12	1.2
Holt Oram	9	0.9
Kabuki	9	0.9
Noonan	47	4.5
Trisomy 21	392	37.9
Turner Syndrome	33	3.2
VATER	9	0.9
VACTERL +/- VATER	56	5.4
Williams	41	4.0
Other Syndromes	68	6.6
Other Autosomal Trisomies	11	1.1
Other X Chromosome Aneuploidy	7	0.7
Other Chromosome Abnormalities^b^	16	1.6
Multiple Syndromes^c^	8	0.8

DGS—DiGeorge Syndrome; VCFS—Velocardiofacial syndrome.

^a^ 11 8p23.1 deletion, 1 deletion/duplication, 1 inversion.

^b^ Ring chromosome, translocation, isochromosome.

^c^ Cases had >1 syndrome.

The nonsyndromic cases are described in Table 3 and the distributions of CHD subtypes (e.g., tetralogy of Fallot, truncus arteriosus) are provided in the S2 Table. The largest subsets of CHDs were CTD (40%) and LVOT (21%). The majority of cases were non-Hispanic White (59%) and male (55%). In addition, cases were predominantly born in the United States (86%), had normal birth weight (77%), did not have extracardiac malformations (76%), and were >1 year of age at recruitment (69%).

**Table 3:** Description of nonsyndromic^a^ cases in the Pediatric Cardiac Genetic Consortium Cohort.

	N^b^ (n = 8,693)	%
CHD Categories		
LAT	779	9.0
CTD	3,500	40.3
AVCD	314	3.6
LVOT	1,834	21.1
RVOT	688	7.9
ASD	770	8.9
Other	808	9.3
Race/Ethnicity		
White	5,110	59.0
Hispanic	1,929	22.3
Black	601	6.9
Asian	574	6.6
Other^c^	447	5.2
Sex		
Male	4,778	55.0
Female	3,914	45.0
Country of Birth		
United States	7,419	85.5
United Kingdom	599	6.9
Other	661	7.6
Birth weight (g)		
Low (<2,500)	1,244	15.8
Normal (2,500–4,000)	6,074	77.1
High (>4,000)	560	7.1
Extracardiac malformations		
Yes	2,113	24.4
No	6,563	75.7
Age at Recruitment		
≤1 year	2,659	30.7
>1 year	6,002	69.3

ASD—atrial septal defect, AVCD—atrioventricular canal defect, CHD—congenital heart defect, CTD—conotruncal heart defect, LAT—laterality disorder, LVOT—left ventricular outflow tract, RVOT—right ventricular outflow tract.

^a^ No recognized clinical syndrome but may have noncardiac anomalies.

^b^ May not sum to total because of missing data.

^c^ Includes Native American/Alaskan, Pacific Islander, and >1 race.

The description of the nonsyndromic cases, by type of CHDs, is provided in Table 4. The distributions of three maternal characteristics, across the six types of CHDs, were significantly different: body mass index (p = 0.002), pre-gestational diabetes (p<0.001), and education (p<0.001). For example, the proportion of cases with an obese mother ranged from 10% (ASD) to 19% (AVCD); the proportion with maternal pre-gestational diabetes ranged from 1% (ASD) to 5% (LAT); and the proportion of cases with a mother with less than a high school education ranged from 4% (AVCD) to 14% (ASD). A significant difference across types of CHDs was also observed for paternal age (p = 0.02) (Table 4). When analyses were restricted to cases ≤1 year at recruitment, similar results were obtained for maternal education and paternal age. However, in this subset, differences were not statistically significant across type of CHD for maternal body mass index or pre-gestational diabetes (S3 Table).

**Table 4:** Demographic, pregnancy, and birth history comparisons of nonsyndromic^a^ cases across major types of congenital heart defect in the Pediatric Cardiac Genetic Consortium Cohort.

All cases Cases ≤1 year	LAT n = 779 n = 220	CTD n = 3,500 n = 1,322	AVCD n = 314 n = 93	LVOT n = 1,834 n = 536	RVOT n = 688 n = 188	ASD n = 770 n = 57	p-value^b^	Total^c^ n = 8,693 n = 2,656
	Mean ± SD							Mean ± SD
Maternal Age	29.3 ± 6.0	29.9 ± 5.8	29.6 ± 5.7	29.9 ± 5.9	29.6 ± 5.8	29.9 ± 6.1	0.15	29.8 ± 5.9
Paternal Age	31.7 ± 6.6	32.7 ± 6.7	32.3 ± 6.7	32.2 ± 6.6	32.3 ± 6.8	32.6 ± 6.7	0.02	32.4 ± 6.6
Mother	N^f^ (%)							N^f^ (%)
Body Mass Index (kg/m^2^)							0.002	
Underweight (<18.5)	56 (8.7)	191 (6.4)	18 (6.6)	85 (5.4)	29 (5.0)	36 (5.7)		459 (6.2)
Normal (18.5-<25)	363 (56.5)	1,814 (60.7)	152 (55.7)	952 (60.0)	338 (57.9)	418 (66.1)		4,458 (60.4)
Overweight (25-<30)	142 (22.1)	587 (19.7)	50 (18.3)	327 (20.6)	135 (23.1)	117 (18.5)		1,494 (20.2)
Obese (≥30)	82 (12.8)	395 (13.2)	53 (19.4)	222 (14.0)	82 (14.0)	61 (9.7)		973 (13.2)
Epilepsy/Seizure							0.09	
Yes	1 (0.1)	19 (0.6)	1 (0.3)	6 (0.3)	8 (1.2)	3 (0.4)		45 (0.5)
No	737 (99.9)	3,323 (99.4)	302 (99.7)	1751 (99.7)	638 (98.8)	718 (99.6)		8,233 (99.5)
Pregestational Diabetes							<0.001	
Yes	34 (4.6)	102 (3.1)	7 (2.3)	27 (1.5)	11 (1.7)	10 (1.4)		213 (2.6)
No	704 (95.4)	3,242 (97.0)	295 (97.7)	1,731 (98.5)	635 (98.3)	710 (98.6)		8,062 (97.4)
Gestational Diabetes							0.58	
Yes	59 (8.1)	243 (7.3)	22 (7.3)	112 (6.4)	47 (7.3)	45 (6.3)		591 (7.2)
No	673 (91.9)	3,083 (92.7)	279 (92.7)	1,640 (93.6)	599 (92.7)	673 (93.7)		7,651 (92.8)
Education^d^							<0.001	
<High school	73 (10.6)	258 (8.6)	12 (4.4)	142 (8.5)	59 (9.5)	91 (13.5)		715 (9.4)
High school	176 (25.6)	659 (21.8)	57 (20.8)	333 (20.0)	140 (22.6)	150 (22.3)		1,673 (21.9)
Partial college	140 (20.4)	723 (24.0)	87 (31.8)	410 (24.6)	146 (23.6)	153 (22.7)		1,832 (23.9)
College or higher	299 (43.5)	1,377 (45.5)	118 (43.1)	779 (46.8)	274 (44.3)	280 (41.5)		3,431 (44.8)
Parity^e^							0.16	
Primiparous	84 (38.7)	623 (47.4)	42 (45.7)	229 (43.1)	78 (43.1)	26 (45.6)		1,182 (44.8)
Multiparous	133 (61.3)	692 (52.6)	50 (54.4)	303 (57.0)	103 (56.9)	31 (54.4)		1,457 (55.2)
Folic Acid^e^							0.36	
Yes	116 (54.0)	769 (58.8)	50 (53.8)	283 (53.6)	101 (56.4)	31 (54.4)		1,468 (55.9)
No	99 (46.1)	539 (41.2)	43 (46.2)	245 (46.4)	78 (43.6)	26 (45.6)		1,157 (44.1)
Smoking^e^							0.45	
Yes	24 (11.1)	119 (9.1)	7 (7.5)	44 (8.3)	11 (6.0)	3 (5.3)		220 (8.3)
No	193 (88.9)	1,196 (91.0)	86 (92.5)	487 (91.7)	171 (94.0)	54 (94.7)		2,419 (91.7)
Alcohol^e^							0.60	
Yes	18 (8.2)	151 (11.5)	8 (8.6)	54 (10.2)	16 (8.8)	5 (8.8)		277 (10.5)
No	201 (91.8)	1,160 (88.5)	85 (91.4)	477 (89.8)	166 (91.2)	52 (91.2)		2,359 (89.5)
Case								
Race/Ethnicity							<0.001	
White	437 (56.5)	2,027 (58.2)	202 (64.3)	1,225 (67.0)	402 (58.4)	404 (52.7)		5,110 (59.0)
Hispanic	179 (23.1)	729 (20.9)	51 (16.2)	391 (21.4)	159 (23.1)	197 (25.7)		1,929 (22.3)
Black	62 (8.0)	251 (7.2)	30 (9.6)	87 (4.8)	55 (8.0)	42 (5.5)		601 (6.9)
Asian	52 (6.7)	268 (7.7)	13 (4.1)	63 (3.4)	35 (5.1)	83 (10.8)		574 (6.6)
Other	44 (5.7)	208 (6.0)	18 (5.7)	63 (3.4)	37 (5.4)	41 (5.4)		447 (5.2)
Sex							<0.001	
Male	445 (57.1)	1,971 (56.3)	122 (38.9)	1,206 (65.8)	337 (49.0)	287 (37.3)		4,778 (55.0)
Female	334 (42.9)	1,528 (43.7)	192 (61.2)	628 (34.2)	351 (51.0)	483 (62.7)		3,914 (45.0)
Birth weight (g)							<0.001	
Low (<2,500)	89 (12.9)	592 (18.6)	41 (14.3)	188 (11.1)	95 (15.5)	127 (18.6)		1,244 (15.8)
Normal (2,500–4,000)	556 (80.7)	2,380 (74.9)	218 (76.2)	1,358 (80.1)	485 (79.0)	516 (75.4)		6,074 (77.1)
High (>4,000)	44 (6.4)	204 (6.4)	27 (9.4)	149 (8.8)	34 (5.5)	41 (6.0)		560 (7.1)
Extracardiac malformations							<0.001	
Yes	393 (50.5)	858 (24.5)	69 (22.0)	369 (20.2)	128 (18.6)	151 (19.7)		2,113 (24.4)
No	386 (49.6)	2,638 (75.5)	245 (78.0)	1,462 (79.9)	560 (81.4)	616 (80.3)		6,563 (75.7)

ASD—atrial septal defect, AVCD—atrioventricular canal defect, CTD—conotruncal heart defect, LAT—laterality disorder, LVOT—left ventricular outflow tract, RVOT—right ventricular outflow tract.

^a^ No recognized clinical syndrome but may have noncardiac anomalies.

^b^ ANOVA test for continuous variables; chi-square test (or Fisher’s exact test when >20% of cells had an expected cell count <5) for categorical variables.

^c^ Includes Other.

^d^ Excluded mothers whose highest education was in the United Kingdom.

^e^ Information only available for cases ≤1 year at recruitment.

^f^ May not sum to total because of missing data.

Although, overall, CHD cases were significantly (p<0.001) more likely to be male (55%) than female, males were predominant in only three of the types of CHDs (LAT, CTD and LVOT) (Table 4). Significant differences across types of CHDs were also observed for case race/ethnicity, birth weight, and extracardiac malformations (p<0.001). For example, the proportion of cases that were non-Hispanic white ranged from 53% (ASD) to 67% (LVOT); the proportion of cases with a low birth weight ranged from 11% (LVOT) to 19% (CTD, ASD); and the proportion of cases that had extracardiac malformations ranged from 19% (RVOT) to 51% (LAT). Similar results were obtained when analyses were restricted cases ≤ 1 year at recruitment (S3 Table).

### Neurodevelopmental outcomes

In nonsyndromic cases, the description of neurodevelopmental outcomes by type of CHDs and the p-values from the unadjusted analyses are provided in Table 5. Differences across types of CHDs were observed for attention deficit hyperactivity disorder (p = 0.03), depression (p = 0.01), developmental delay (p = 0.003), learning disability (p<0.001), repeated grade (p<0.001), and the composite neurodevelopmental outcome variable (p<0.001). The frequencies of these outcomes were highest for cases with RVOT (attention deficit hyperactivity disorder, 10%; depression, 10%) or AVCD (developmental delay, 17%; learning disability, 21%; repeated grade, 21%; composite measure, 28%) and lowest for cases with ASD (5%, 6%, 8%, 11%, 13%, and 10%, respectively). Results were similar in the adjusted analyses; however, differences across types of CHDs were no longer significant for attention deficit hyperactivity disorder and depression, and the adjusted model did not converge for autism spectrum and other neurodevelopmental outcomes.

**Table 5:** Neurodevelopmental outcomes across major types of congenital heart defect for nonsyndromic^a^ cases ≥ 5 years old in the Pediatric Cardiac Genetic Consortium Cohort.

Cases ≥5 year	LAT n = 412	CTD n = 1,720	AVCD n = 145	LVOT n = 1,045	RVOT n = 380	ASD n = 448	p-value^b^	Total^c^ n = 4,587
	N^e^ (%)							N^e^| (%)
Attention deficit hyperactivity disorder							0.03	
Yes	33 (8.0)	163 (9.5)	12 (8.4)	100 (9.7)	37 (9.8)	21 (4.7)		403 (8.9)
No	378 (92.0)	1,545 (90.5)	131 (91.6)	932 (90.3)	341 (90.2)	423 (95.3)		4,148 (91.1)
Anxiety							0.76	
Yes	37 (9.0)	169 (9.9)	13 (9.0)	86 (8.3)	38 (10.0)	38 (8.5)		414 (9.1)
No	373 (91.0)	1,538 (90.1)	132 (91.0)	950 (91.7)	341 (90.0)	409 (91.5)		4,146 (90.9)
Autism Spectrum							0.07	
Yes	3 (0.7)	41 (2.4)	2 (1.4)	22 (2.1)	5 (1.3)	3 (0.7)		86 (1.9)
No	408 (99.3)	1,673 (97.6)	143 (98.6)	1,017 (97.9)	373 (98.7)	443 (99.3)		4,483 (98.1)
Behavioral							0.09	
Yes	15 (3.7)	72 (4.2)	3 (2.1)	26 (2.5)	10 (2.6)	10 (2.2)		150 (3.3)
No	395 (96.3)	1,634 (95.8)	142 (97.9)	1,011 (97.5)	369 (97.4)	437 (97.8)		4,409 (96.7)
Depression							0.01	
Yes	29 (7.1)	116 (6.8)	11 (7.6)	45 (4.3)	36 (9.5)	26 (5.8)		286 (6.3)
No	381 (92.9)	1,591 (93.2)	134 (92.4)	996 (95.7)	344 (90.5)	422 (94.2)		4,280 (93.7)
Developmental Delay							0.003	
Yes	63 (15.4)	240 (14.0)	25 (17.2)	118 (11.4)	53 (14.1)	37 (8.3)		576 (12.6)
No	346 (84.6)	1,470 (86.0)	120 (82.8)	992 (88.7)	324 (85.9)	411 (91.7)		3,989 (87.4)
Learning Disability							<0.001	
Yes	85 (20.8)	327 (19.2)	30 (21.4)	166 (16.0)	81 (21.4)	49 (11.0)		796 (17.5)
No	324 (79.2)	1,376 (80.8)	110 (78.6)	874 (84.0)	297 (78.6)	398 (89.0)		3,755 (82.5)
Mental Retardation							0.35	
Yes	4 (1.0)	37 (2.2)	2 (1.4)	16 (1.5)	6 (1.6)	4 (0.9)		72 (1.6)
No	406 (99.0)	1,669 (97.8)	143 (98.6)	1,022 (98.5)	371 (98.4)	442 (99.1)		4,483 (98.4)
Obsessive-compulsive disorder							0.38	
Yes	5 (1.2)	38 (2.2)	2 (1.4)	15 (1.4)	10 (2.7)	6 (1.3)		78 (1.7)
No	406 (98.8)	1,675 (97.8)	143 (98.6)	1,025 (98.6)	368 (97.4)	442 (98.7)		4,492 (98.3)
Repeated Grade							<0.001	
Yes	57 (14.7)	246 (15.2)	29 (21.3)	99 (10.1)	58 (16.4)	42 (10.3)		567 (13.2)
No	330 (85.3)	1,376 (84.8)	107 (78.7)	881 (89.9)	296 (83.6)	367 (89.7)		3,727 (86.8)
Seizure Disorder							0.19	
Yes	18 (4.4)	53 (3.1)	5 (3.4)	34 (3.3)	14 (3.7)	6 (1.3)		143 (3.1)
No	391 (95.6)	1,657 (96.9)	140 (96.7)	1,005 (96.7)	364 (96.3)	441 (98.7)		4,420 (96.9)
Speech Problem							0.18	
Yes	64 (15.6)	271 (15.8)	20 (13.8)	152 (14.6)	49 (12.9)	50 (11.2)		662 (14.5)
No	346 (84.4)	1,442 (84.2)	125 (86.2)	889 (85.4)	330 (87.1)	398 (88.8)		3,909 (85.5)
Other							0.46	
Yes	9 (2.2)	40 (2.3)	2 (1.4)	18 (1.7)	7 (1.9)	4 (0.9)		83 (1.8)
No	402 (97.8)	1,673 (97.7)	143 (98.6)	1,021 (98.3)	372 (98.2)	444 (99.1)		4,488 (98.2)
Composite^d^							<0.001	
Yes	114 (27.7)	425 (24.7)	41 (28.3)	215 (20.6)	106 (27.9)	62 (13.8)		1,044 (22.8)
No	298 (72.3)	1,295 (75.3)	104 (71.7)	830 (79.4)	274 (72.1)	386 (86.2)		3,543 (77.2)

ASD—atrial septal defect, AVCD—atrioventricular canal defect, CTD—conotruncal heart defect, LAT—laterality disorder, LVOT—left ventricular outflow tract, RVOT—right ventricular outflow tract.

^a^ No recognized clinical syndrome but may have noncardiac anomalies.

^b^ Unadjusted logistic regression.

^c^ Includes Other.

^d^ Composite variable indicating a positive parental report of autism, developmental delay, learning disability, or mental retardation.

^e^ May not sum to total because of missing data.

### CTD and LVOT subtypes

Analyses were repeated to assess differences across subtypes within CTDs and LVOTs (S4 and S5 Tables). Given the relatively small numbers of subtypes of CHDs in these two groups, only unadjusted analyses were conducted. Significant differences across the four subtypes of CTDs were observed for maternal body mass index (p = 0.03), pre-gestational diabetes (p = 0.04), and parity (p = 0.01). Significant difference were also observed for infant sex (p<0.001), race/ethnicity (p<0.001), birth weight (p<0.001), extracardiac malformations (p<0.001), and parent-reported anxiety (p = 0.03).

Across the three subtypes of LVOTs, significant differences were observed for maternal education (p = 0.005), infant sex (p<0.001), race/ethnicity (p<0.001), birth weight (p = 0.008), extracardiac malformations (p = 0.04), and several neurodevelopmental outcomes. In general, adverse neurodevelopmental outcomes appeared to be reported more frequently by parents of hypoplastic left heart syndrome (HLHS) cases than by parents of aortic valve disease and coarctation of the aorta cases.

## Discussion

Between 2010 and 2014, the PCGC recruited over 9,000 families with a child affected by a CHD. This cohort is one of only a few large contemporary CHD cohorts that can be used to study the genetic basis of the causes and consequences of these common and serious birth defects. The PCGC has established data sharing plans (https://benchtobassinet.com/ForResearchers/B2BDataSharingPlan.aspx), which include data access through dbGap (dbGAP Accession: phs000571.v3.p2) and has established a process for proposing ancillary studies that make use of biospecimens. Hence, the PCGC cohort provides a valuable resource for the research community. This paper, in conjunction with an earlier report describing the rationale and design of the PCGC [11], provides investigators with details that should help to inform their study design (e.g., phenotype selection), analytic plan (e.g., power, subgroup analyses), and interpretation of study results (e.g., study limitations).

As enrollment for the PCGC cohort was through tertiary/quaternary medical centers, it was skewed toward cases with more severe forms of CHD. However, the cohort includes both cases with complex and cases with simple lesions, so it is broadly representative of the spectrum of clinically significant CHDs. For example, the low frequency of males among cases with ASDs and AVCDs is consistent with previous findings [10, 16–18]. As recruitment was center-specific, it is possible that differences in recruitment might have introduced some selection bias. For instances, the proportion of cases in the PCGC cohort with a genetic diagnosis (11%) is low compared to population-based estimates (~20%) [19]. This likely reflects the PCGC recruitment priorities (e.g., nonsyndromic over syndromic) and it is possible that some centers may have recruited a lower proportion of syndromic cases than other centers.

Because cases of all ages were eligible and information was collected via subject or family interviews, these data are subject to recall errors. Recall error may account for differences in estimates obtained from the PCGC and from other studies. For example, this issue might explain why the proportion of mothers of PCGC cases who reported that they took folic acid *prior* to becoming pregnant is relatively high (56%), compared to estimates based on women who were pregnant or of child-bearing age (<45%) [20–22]. Additionally, because neurodevelopmental outcomes in cases were reported by parents, the reported frequencies may not reflect the distribution of neurodevelopmental outcomes in the general CHD population [23].

The PCGC did not conduct a case-control study. Since there is no comparable control group, the cohort cannot be used to study non-genetic risk factors for CHDs and CHD outcomes. However, differences in the distribution of known CHD risk factors (e.g., race/ethnicity, maternal pre-gestational diabetes) across types and subtypes of CHDs provide potentially important insights into the data. For example, in the PCGC cohort, the proportion of cases of Hispanic ethnicity differs across types of CHDs. As similar differences have been observed in population-based epidemiologic studies [24, 25], this may reflect true underlying differences in the risk factor profiles of the different CHDs. Nonetheless, these differences might also be artificial. For example, these differences may be a result of lesion-specific differences in survival by ethnicity [26] or differences in ascertainment by ethnicity and/or type of CHDs. Either way, investigators need to be aware of these differences, since they may influence the results for studies of genetic variants that differ in frequency across ethnic groups.

In summary, we provide a description of the distribution of key variables in the PCGC cohort and identified differences in the distribution of certain characteristics across types and subtypes of CHDs. This information will help inform future genomic studies on the etiology and neurodevelopmental outcomes across types and subtypes of CHDs in the PGCG cohort.

## Supporting information

S1 Table Type of congenital heart defect among cases with trisomy 21 or DiGeorge syndrome/Velocardiofacial syndrome/22q11.2 deletion in the Pediatric Cardiac Genetic Consortium Cohort. ASD—atrial septal defect, AVCD—atrioventricular canal defect, CTD—conotruncal heart defect, DGS—DiGeorge syndrome, LAT—laterality disorder, LVOT—left ventricular outflow tract, RVOT—right ventricular outflow tract, VCFS—velocardiofacial syndrome.
(DOCX)

S2 Table Congenital heart defect (CHD) phenotypes within each type of CHD in nonsyndromic^a^ cases in the Pediatric Cardiac Genetic Consortium Cohort. ASD—atrial septal defect, AVCD—atrioventricular canal defect, CTD—conotruncal heart defect, LAT—laterality disorder, LVOT—left ventricular outflow tract, RVOT—right ventricular outflow tract.
(DOCX)

S3 Table Demographic, pregnancy, and birth history comparisons of nonsyndromic^a^ cases across major types of congenital heart defect for cases ≤ 1 year of age at recruitment in the Pediatric Cardiac Genetic Consortium Cohort. ASD—atrial septal defect, AVCD—atrioventricular canal defect, CTD—conotruncal heart defect, LAT—laterality disorder, LVOT—left ventricular outflow tract, RVOT—right ventricular outflow tract.
(DOCX)

S4 Table Demographic, pregnancy, and birth history comparisons of nonsyndromic^a^ cases across major CTD and LVOT subtypes in the Pediatric Cardiac Genetic Consortium Cohort. AVD—aortic valve disease (aortic stenosis, bicuspid aortic valve), COA—coarctation of the aorta, CTD—conotruncal heart defect, DORV—double outlet right ventricle, D-TGA—D-transposition of the great arteries, HLHS—hypoplastic left heart syndrome, LVOT—left ventricular outflow tract, TOF—tetralogy of Fallot, VSD—ventricular septal defect.
(DOCX)

S5 Table Neurodevelopmental outcomes across major CTD and LVOT subtypes for nonsyndromic^a^ cases ≥5 years in the Pediatric Cardiac Genetic Consortium Cohort. AVD—aortic valve disease (aortic stenosis, bicuspid aortic valve), COA—coarctation of the aorta, CTD—conotruncal heart defect, DORV—double outlet right ventricle, D-TGA—D-transposition of the great arteries, HLHS—hypoplastic left heart syndrome, LVOT—left ventricular outflow tract, TOF—tetralogy of Fallot, VSD–ventricular septal defect.
(DOCX)

S1 Dataset Anonymized data set for replicating the results from the Pediatric Cardiac Genetic Consortium cohort description. (XLSX)
