# Supplementary material for: The Congenital Heart Disease Genetic Network Study: Cohort description
Source: PLoS One. 2018 Jan 19;13(1):e0191319. doi: 10.1371/journal.pone.0191319 (PMC5774789; doi:10.1371/journal.pone.0191319)
Supplement: S1 Table — ASD—atrial septal defect, AVCD—atrioventricular canal defect, CTD—conotruncal heart defect, DGS—DiGeorge syndrome, LAT—laterality disorder, LVOT—left ventricular outflow tract, RVOT—right ventricular outflow tract, VCFS—velocardiofacial syndrome. (DOCX) [file pone.0191319.s001.docx]

S1 Table. Type of congenital heart defect among

cases with trisomy 21 or DiGeorge syndrome/Velocardiofacial
syndrome/22q11.2 deletion in the Pediatric Cardiac Genetic
Consortium Cohort

|  | Trisomy 21 | | | DGS/VCFS/22q11.2  deletion | |
| --- | --- | --- | --- | --- | --- |
|  |  |  |  | |  |
|  | N | % | N | | % |
|  |  |  |  | |  |
| LAT | 3 | 0.8 | 4 | | 1.6 |
| CTD | 138 | 35.2 | 240 | | 95.6 |
| AVCD | 205 | 52.3 | 0 | | 0.0 |
| LVOT | 4 | 1.0 | 2 | | 0.8 |
| RVOT | 0 | 0.0 | 1 | | 0.4 |
| ASD | 25 | 6.4 | 2 | | 0.8 |
| Other | 17 | 4.3 | 2 | | 0.8 |

ASD - atrial septal defect, AVCD - atrioventricular canal defect,
CTD – conotruncal heart defect, DGS – DiGeorge syndrome,
LAT – laterality disorder, LVOT - left ventricular outflow tract,
RVOT - right ventricular outflow tract, VCFS – velocardiofacial
syndrome.
