# Supplementary material for: The Congenital Heart Disease Genetic Network Study: Cohort description
Source: PLoS One. 2018 Jan 19;13(1):e0191319. doi: 10.1371/journal.pone.0191319 (PMC5774789; doi:10.1371/journal.pone.0191319)
Supplement: S2 Table — ASD—atrial septal defect, AVCD—atrioventricular canal defect, CTD—conotruncal heart defect, LAT—laterality disorder, LVOT—left ventricular outflow tract, RVOT—right ventricular outflow tract. (DOCX) [file pone.0191319.s002.docx]

S2 Table. Congenital heart defect (CHD) phenotypes within each CHD type in nonsyndromic^a^
cases in the Pediatric Cardiac Genetic Consortium Cohort

| CHD Phenotypes | | N | % |
| --- | --- | --- | --- |
| LAT | |  |  |
| Laterality disorder | | 779 | 100 |
| CTD | |  |  |
|  | Tetralogy of Fallot | 1,282 | 36.6 |
|  | Truncus arteriosus | 141 | 4.0 |
|  | Interrupted aortic arch | 45 | 1.3 |
|  | Double outlet right ventricle | 304 | 8.7 |
|  | D-transposition of the great arteries | 708 | 20.2 |
|  | Isolated ventricular septal defect^b^ | 621 | 17.7 |
|  | Isolated aortic arch anomaly | 121 | 3.5 |
|  | Other | 278 | 7.9 |
| AVCD | |  |  |
| Complete AVCD | | 129 | 41.1 |
| Transitional AVCD | | 71 | 22.6 |
| Primum ASD | | 99 | 31.5 |
| Other | | 15 | 4.8 |
| LVOT | |  |  |
|  | Aortic stenosis | 422 | 23.0 |
|  | Coarctation of the aorta | 604 | 32.9 |
|  | Hypoplastic left heart syndrome | 454 | 24.8 |
|  | Bicuspid aortic valve, not coded for stenosis | 190 | 10.4 |
|  | Other | 164 | 9.0 |
| RVOT (with normally related great arteries) | |  |  |
|  | Tricuspid valve atresia | 144 | 20.9 |
|  | Pulmonary valve stenosis | 336 | 48.8 |
|  | Pulmonary valve atresia | 208 | 30.2 |
| ASD | |  |  |
|  | Secundum | 631 | 82.0 |
|  | Sinus venosus ASD | 122 | 15.8 |
|  | NOS – Not otherwise specified | 17 | 2.2 |
| Other | |  |  |
|  | Total anomalous pulmonary venous return | 105 | 13.0 |
|  | Partial anomalous pulmonary venous return | 65 | 8.0 |
|  | Coronary artery anomaly | 99 | 12.3 |
|  | Ebstein/abnormal TV | 145 | 18.0 |
|  | Ventricular septal defect (muscular) | 94 | 11.6 |
|  | Patent ductus arteriosus | 100 | 12.4 |
|  | Complex | 86 | 10.6 |
|  | Other | 114 | 14.1 |

ASD - atrial septal defect, AVCD - atrioventricular canal defect, CTD – conotruncal heart defect, LAT – laterality disorder, LVOT - left ventricular outflow tract, RVOT - right ventricular outflow tract.

^a^ No recognized clinical syndrome but may have noncardiac anomalies.

^b^ Conoventricular, conoseptal hypoplasia and posterior malalignment type ventricular septal defects.
