# Supplementary material for: The Congenital Heart Disease Genetic Network Study: Cohort description
Source: PLoS One. 2018 Jan 19;13(1):e0191319. doi: 10.1371/journal.pone.0191319 (PMC5774789; doi:10.1371/journal.pone.0191319)
Supplement: S3 Table — ASD—atrial septal defect, AVCD—atrioventricular canal defect, CTD—conotruncal heart defect, LAT—laterality disorder, LVOT—left ventricular outflow tract, RVOT—right ventricular outflow tract. (DOCX) [file pone.0191319.s003.docx]

S3 Table. Demographic, pregnancy, and birth history comparisons of nonsyndromic^a^ cases across major types of congenital heart defect for cases
< 1 year of age at recruitment in the Pediatric Cardiac Genetic Consortium Cohort

|  | **LAT** | **CTD** | **AVCD** | **LVOT** | **RVOT** | **ASD** | p-value^b^ | **Total**^c^ |
| --- | --- | --- | --- | --- | --- | --- | --- | --- |
| Cases <1 year | n=220 | n=1,322 | n=93 | n=536 | n=188 | n=57 |  | n=2,656 |
|  | Mean + SD | | | | | |  | Mean + SD |
|  |  |  |  |  |  |  |  |  |
| Maternal Age | 30.0 + 6.1 | 30.6 + 5.6 | 30.6 + 5.8 | 30.0 + 5.9 | 30.3 + 5.7 | 29.2 + 6.2 | 0.14 | 30.3 + 5.8 |
| Paternal Age | 32.1 + 6.8 | 33.2 + 6.8 | 33.2 + 6.8 | 32.1 + 6.7 | 33.2 + 7.3 | 31.9 + 7.2 | 0.04 | 32.8 + 6.9 |
|  |  |  |  |  |  |  |  |  |
| Mother | N^e^ (%) | | | | | |  | N# (%) |
| Body Mass Index (kg/m^2^) |  |  |  |  |  |  | 0.33 |  |
| Underweight (<18.5) | 11 (5.6) | 62 (5.0) | 2 (2.3) | 19 (3.8) | 5 (2.8) | 3 (6.0) |  | 113 (4.6) |
| Normal (18.5-<25) | 97 (49.5) | 630 (51.1) | 41 (47.7) | 260 (52.1) | 88 (49.7) | 19 (38.0) |  | 1,252 (50.5) |
| Overweight (25-<30) | 57 (29.1) | 315 (25.6) | 19 (22.1) | 118 (23.7) | 44 (24.9) | 20 (40.0) |  | 626 (25.5) |
| Obese (>30) | 31 (15.8) | 225 (18.3) | 24 (27.9) | 102 (20.4) | 40 (22.6) | 8 (16.0) |  | 477 (19.4) |
| Epilepsy/Seizure |  |  |  |  |  |  | 0.49† |  |
| Yes | 0 (0.0) | 6 (0.5) | 0 (0.0) | 1 (0.2) | 2 (1.1) | 0 (0.0) |  | 11 (0.4) |
| No | 219 (100.0) | 1,310 (99.5) | 93 (100.0) | 530 (99.8) | 184 (98.9) | 57 (100.0) |  | 2,631 (99.6) |
| Pregestational Diabetes |  |  |  |  |  |  | 0.28 |  |
| Yes | 14 (6.4) | 54 (4.1) | 5 (5.4) | 13 (2.5) | 7 (3.8) | 3 (5.3) |  | 107 (4.1) |
| No | 205 (93.6) | 1,264 (95.9) | 88 (94.6) | 518 (97.6) | 179 (96.2) | 54 (94.7) |  | 2,534 (96.0) |
| Gestational Diabetes |  |  |  |  |  |  | 0.2 |  |
| Yes | 26 (11.9) | 123 (9.4) | 6 (6.5) | 37 (7.0) | 19 (10.2) | 6 (10.5) |  | 246 (9.3) |
| No | 193 (88.1) | 1,193 (90.7) | 87 (93.6) | 495 (93.1) | 168 (89.8) | 51 (89.5) |  | 2,398 (90.7) |
| Education^d^ |  |  |  |  |  |  | 0.007 |  |
| <High school | 25 (12.3) | 115 (9.8) | 2 (2.5) | 47 (9.6) | 13 (7.7) | 8 (16.0) |  | 242 (10.1) |
| High school | 48 (23.7) | 201 (17.1) | 16 (19.8) | 93 (19.0) | 30 (17.8) | 11 (22.0) |  | 450 (18.8) |
| Partial college | 41 (20.2) | 281 (23.9) | 30 (37.0) | 126 (25.8) | 42 (24.9) | 15 (30.0) |  | 588 (24.6) |
| College or higher | 89 (43.8) | 581 (49.3) | 33 (40.7) | 223 (45.6) | 84 (49.7) | 16 (32.0) |  | 1,112 (46.5) |

ASD - atrial septal defect, AVCD - atrioventricular canal defect, CTD – conotruncal heart defect, LAT – laterality disorder, LVOT - left ventricular outflow tract, RVOT - right ventricular outflow tract.

^a^ No recognized clinical syndrome but may have noncardiac anomalies.

^b^ ANOVA test for continuous variables; chi-square test (or Fisher’s exact test when >20% of cells had an expected cell count <5) for categorical variables.

^c^ Includes Other.
^d^ Excluded mothers whose highest education was in the United Kingdom.
^e^ May not sum to total because of missing data.

S3 Table (cont’d). Demographic, pregnancy, and birth history comparisons of nonsyndromic^a^ cases across major types of congenital heart defect for cases < 1 year of age at recruitment in the Pediatric Cardiac Genetic Consortium Cohort

|  | | **LAT** | **CTD** | **AVCD** | **LVOT** | **RVOT** | **ASD** | | p-value^b^ | | **Total**^c^ |
| --- | --- | --- | --- | --- | --- | --- | --- | --- | --- | --- | --- |
| Cases <1 year | | n=220 | n=1,322 | n=93 | n=536 | n=188 | n=57 | |  |  | n=2,656 |
| Case | | N^e^ (%) | | | | | | | |  | N# (%) |
| Race/Ethnicity | |  |  |  |  |  | |  | <0.001 | |  |
| White | | 96 (43.8) | 692 (52.5) | 52 (55.9) | 334 (62.6) | 107 (56.9) | 20 (35.7) | |  | | 1,397 (52.7) |
| Hispanic | | 71 (32.4) | 339 (25.7) | 21 (22.6) | 126 (23.6) | 45 (23.9) | 25 (44.6) | |  | | 726 (27.4) |
| Black | | 16 (7.3) | 88 (6.7) | 11 (11.8) | 28 (5.2) | 9 (4.8) | 2 (3.6) | |  | | 173 (6.5) |
| Asian | | 13 (5.9) | 99 (7.5) | 5 (5.4) | 17 (3.2) | 16 (8.5) | 5 (8.9) | |  | | 162 (6.1) |
| Other | | 23 (10.5) | 101 (7.7) | 4 (4.3) | 29 (5.4) | 16 (8.5) | 4 (7.1) | |  | | 192 (7.3) |
| Sex | |  |  |  |  |  |  | | <0.001 | |  |
| Male | | 125 (56.8) | 761 (57.6) | 36 (38.7) | 330 (61.6) | 96 (51.1) | 24 (42.1) | |  | | 1,499 (56.4) |
| Female | | 95 (43.2) | 561 (42.4) | 57 (61.3) | 206 (38.4) | 92 (48.9) | 33 (57.9) | |  | | 1,160 (43.6) |
| Birth weight (g) | |  |  |  |  |  |  | | 0.038 | |  |
| Low (<2,500) | | 33 (15.2) | 242 (18.4) | 19 (20.7) | 70 (13.1) | 31 (16.7) | 9 (15.8) | |  | | 449 (17.0) |
| Normal (2,500-4,000) | | 176 (81.1) | 1,012 (77.1) | 64 (69.6) | 432 (81.1) | 146 (78.5) | 42 (73.7) | |  | | 2,050 (77.7) |
| High (>4,000) | | 8 (3.7) | 59 (4.5) | 9 (9.8) | 31 (5.8) | 9 (4.8) | 6 (10.5) | |  | | 140 (5.3) |
| Extracardiac malformations | | |  |  |  |  |  | | | <0.001 |  |
| Yes | 137 (62.3) | | 368 (27.9) | 29 (31.2) | 131 (24.4) | 36 (19.2) | 20 (35.7) | |  | | 767 (28.9) |
| No | | 83 (37.7) | 953 (72.1) | 64 (68.8) | 405 (75.6) | 152 (80.9) | 36 (64.3) | |  | | 1,886 (71.1) |

ASD - atrial septal defect, AVCD - atrioventricular canal defect, CTD – conotruncal heart defect, LAT – laterality disorder, LVOT - left ventricular outflow tract, RVOT - right ventricular outflow tract.

^a^ No recognized clinical syndrome but may have noncardiac anomalies.

^b^ ANOVA test for continuous variables; chi-square test (or Fisher’s exact test when >20% of cells had an expected cell count <5) for categorical variables.

^c^ Includes Other.
^d^ Excluded mothers whose highest education was in the United Kingdom.
^e^ May not sum to total because of missing data.
