# Supplementary material for: The Congenital Heart Disease Genetic Network Study: Cohort description
Source: PLoS One. 2018 Jan 19;13(1):e0191319. doi: 10.1371/journal.pone.0191319 (PMC5774789; doi:10.1371/journal.pone.0191319)
Supplement: S4 Table — AVD—aortic valve disease (aortic stenosis, bicuspid aortic valve), COA—coarctation of the aorta, CTD—conotruncal heart defect, DORV—double outlet right ventricle, D-TGA—D-transposition of the great arteries, HLHS—hypoplastic left heart syndrome, LVOT—left ventricular outflow tract, TOF—tetralogy of Fallot, VSD—ventricular septal defect. (DOCX) [file pone.0191319.s004.docx]

| S4 Table. Demographic, pregnancy, and birth history comparisons of nonsyndromic^a^ cases across major CTD and LVOT subtypes in the Pediatric Cardiac Genetic Consortium Cohort | | | | | | | | | | | | | | | | | |
| --- | --- | --- | --- | --- | --- | --- | --- | --- | --- | --- | --- | --- | --- | --- | --- | --- | --- |
|  | **CTD** | | | | | | |  | | **LVOT** | | | | | | | |
|  | **DORV** | **D-TGA** | **TOF** | **VSD**^b^ | | p-value^c^ | |  | | **AVD** | | **COA** | | **HLHS** | | p-value^c^ | |
| All cases | n=304 | n=708 | n=1,282 | n=621 | |  |  |  | | n=612 | | n=604 | | n=454 | |  |  |
| Cases <1 year | n=127 | n=245 | n=454 | n=263 | |  |  |  | | n=63 | | n=220 | | n=200 | |  |  |
|  | Mean + SD or Median (IQR) | | | | |  | |  | | Mean + SD | | | | | |  | |
| Maternal Age | 29.9 + 5.9 | 30.0 + 5.6 | 29.7 + 5.7 | 29.9 + 6.1 | | 0.76 | |  | | 30.1 + 5.7 | | 30.2 + 5.8 | | 29.6 + 6.0 | | 0.25 | |
| Paternal Age | 32.5 + 6.6 | 32.7 + 6.2 | 32.5 + 6.6 | 33.0 + 7.3 | | 0.54^g^ | |  | | 32.4 + 6.1 | | 32.6 + 6.5 | | 31.8 + 6.3 | | 0.13 | |
| Maternal | N^f^ (%) | | | | |  | |  | | N^f^ (%) | | | | | |  | |
| Body Mass Index (kg/m^2^) |  |  |  | |  | 0.03 | |  | |  | |  | |  | | 0.31 | |
| Underweight (<18.5) | 14 (5.6) | 35 (7.2) | 65 (6.1) | | 30 (5.6) |  | |  | | 31 (6.0) | | 29 (5.5) | | 13 (3.2) | |  | |
| Normal (18.5-<25) | 138 (55.4) | 324 (66.3) | 651 (61.1) | | 337 (62.4) |  | |  | | 314 (61.2) | | 306 (58.0) | | 248 (60.6) | |  | |
| Overweight (25-<30) | 60 (24.1) | 91 (18.6) | 202 (19.0) | | 106 (19.6) |  | |  | | 96 (18.7) | | 116 (22.0) | | 94 (23.0) | |  | |
| Obese (>30) | 37 (14.9) | 39 (8.0) | 147 (13.8) | | 67 (12.4) |  | |  | | 72 (14.0) | | 77 (14.6) | | 54 (13.2) | |  | |
| Epilepsy/Seizure |  |  |  | |  | 0.20 | |  | |  | |  | |  | | 0.52 | |
| Yes | 1 (0.3) | 1 (0.2) | 11 (0.9) | | 4 (0.7) |  | |  | | 2 (0.3) | | 1 (0.2) | | 3 (0.7) | |  | |
| No | 293 (99.7) | 670 (99.9) | 1,196 (99.1) | | 590 (99.3) |  | |  | | 580 (99.7) | | 578 (99.8) | | 441 (99.3) | |  | |
| Pregestational Diabetes |  |  |  | |  | 0.04 | |  | |  | |  | |  | | 0.16 | |
| Yes | 15 (5.1) | 16 (2.4) | 38 (3.2) | | 11 (1.9) |  | |  | | 14 (2.4) | | 6 (1.0) | | 6 (1.4) | |  | |
| No | 279 (94.9) | 658 (97.6) | 1,168 (96.9) | | 583 (98.2) |  | |  | | 569 (97.6) | | 574 (99.0) | | 438 (98.7) | |  | |
| Gestational Diabetes |  |  |  | |  | 0.06 | |  | |  | |  | |  | | 0.38 | |
| Yes | 27 (9.4) | 35 (5.2) | 90 (7.5) | | 35 (5.9) |  | |  | | 43 (7.4) | | 33 (5.7) | | 25 (5.6) | |  | |
| No | 261 (90.6) | 636 (94.8) | 1,112 (92.5) | | 558 (94.1) |  | |  | | 536 (92.6) | | 546 (94.3) | | 418 (94.4) | |  | |
| Education^d^ |  |  |  | |  | 0.16 | |  | |  | |  | |  | | 0.005 | |
| <High school | 24 (9.7) | 35 (5.8) | 96 (8.7) | | 52 (9.8) |  | |  | | 43 (7.5) | | 40 (7.3) | | 39 (9.5) | |  | |
| High school | 53 (21.5) | 144 (23.7) | 259 (23.3) | | 105 (19.8) |  | |  | | 113 (19.8) | | 98 (18.0) | | 91 (22.3) | |  | |
| Partial college | 69 (27.9) | 143 (23.6) | 255 (23.0) | | 128 (24.2) |  | |  | | 144 (25.2) | | 113 (20.7) | | 115 (28.1) | |  | |
| College or higher | 101 (40.9) | 285 (47.0) | 500 (45.1) | | 245 (46.2) |  | |  | | 272 (47.6) | | 294 (53.9) | | 164 (40.1) | |  | |
| Parity^e^ |  |  |  | |  | 0.01 |  | |  | |  | |  | | 0.80 | |  |
| Primiparous | 62 (48.8) | 95 (38.9) | 232 (51.2) | | 134 (51.7) |  |  | | 25 (39.7) | | 97 (44.3) | | 84 (42.4) | |  | |  |
| Multiparous | 65 (51.2) | 149 (61.1) | 221 (48.8) | | 125 (48.3) |  |  | | 38 (60.3) | | 122 (55.7) | | 114 (57.6) | |  | |  |

AVD – aortic valve disease (aortic stenosis, bicuspid aortic valve), COA - coarctation of the aorta, CTD – conotruncal heart defect, DORV - double outlet right ventricle, D-TGA – D-transposition of the great arteries, HLHS – hypoplastic left heart syndrome , LVOT – left ventricular outflow tract, TOF – tetralogy of Fallot, VSD – ventricular septal defect.
^a^ No recognized clinical syndrome but may have noncardiac anomalies.

^b^ Conoventricular, conoseptal hypoplasia and posterior malalignment type ventricular septal defects.

^c^ ANOVA test for continuous variables; chi-square test (or Fisher’s exact test when >20% of cells had an expected cell count <5) for categorical variables.
^d^ Excluded mothers whose highest education was in the United Kingdom.
^e^ Information only available for cases <1 year at recruitment.
^f^ May not sum to total because of missing data.

^g^ Used Welch’s ANOVA because due to heterogeneity of variance.

| S4 Table (cont’d). Demographic, pregnancy, and birth history comparisons of nonsyndromic^a^ cases across major CTD and LVOT subtypes in the Pediatric Cardiac Genetic Consortium Cohort | | | | | | | | | | |
| --- | --- | --- | --- | --- | --- | --- | --- | --- | --- | --- |
|  | **CTD** | | | | |  | **LVOT** | | | |
|  | **DORV** | **D-TGA** | **TOF** | **VSD**^b^ | p-value^c^ |  | **AVD** | **COA** | **HLHS** | p-value^c^ |
| All cases | n=304 | n=708 | n=1,282 | n=621 |  |  | n=612 | n=604 | n=454 |  |
| Cases <1 year | n=127 | n=245 | n=454 | n=263 |  |  | n=63 | n=220 | n=200 |  |
| Maternal | N^f^ (%) | | | |  |  | N^f^ (%) | | |  |
| Folic Acid^e^ |  |  |  |  | 0.18 |  |  |  |  | 0.13 |
| Yes | 66 (52.0) | 152 (62.8) | 266 (58.9) | 160 (62.0) |  |  | 28 (45.2) | 127 (58.5) | 102 (51.8) |  |
| No | 61 (48.0) | 90 (37.2) | 186 (41.2) | 98 (38.0) |  |  | 34 (54.8) | 90 (41.5) | 95 (48.2) |  |
| Smoking^e^ |  |  |  |  | 0.81 |  |  |  |  | 0.43 |
| Yes | 8 (6.3) | 21 (8.6) | 40 (8.8) | 20 (7.7) |  |  | 7 (11.1) | 14 (6.4) | 17 (8.6) |  |
| No | 119 (93.7) | 222 (91.4) | 414 (91.2) | 239 (92.3) |  |  | 56 (88.9) | 204 (93.6) | 181 (91.4) |  |
| Alcohol^e^ |  |  |  |  | 0.54 |  |  |  |  | 0.97 |
| Yes | 18 (14.2) | 26 (10.7) | 58 (12.8) | 26 (10.0) |  |  | 6 (9.5) | 22 (10.1) | 21 (10.6) |  |
| No | 109 (85.8) | 217 (89.3) | 395 (87.2) | 233 (90.0) |  |  | 57 (90.5) | 196 (89.9) | 177 (89.4) |  |
| Case |  |  |  |  |  |  |  |  |  |  |
| Sex |  |  |  |  | <0.001 |  |  |  |  | <0.001 |
| Male | 188 (61.8) | 444 (62.7) | 716 (55.9) | 320 (51.5) |  |  | 442 (72.2) | 375 (62.1) | 297 (65.4) |  |
| Female | 116 (38.2) | 264 (37.3) | 565 (44.1) | 301 (48.5) |  |  | 170 (27.8) | 229 (37.9) | 157 (34.6) |  |
| Race/Ethnicity |  |  |  |  | <0.001 |  |  |  |  | <0.001 |
| White | 148 (48.7) | 475 (67.4) | 765 (60.1) | 277 (44.8) |  |  | 432 (70.7) | 426 (70.8) | 275 (60.7) |  |
| Hispanic | 70 (23.0) | 126 (17.9) | 246 (19.3) | 169 (27.4) |  |  | 125 (20.5) | 112 (18.6) | 105 (23.2) |  |
| Black | 34 (11.2) | 29 (4.1) | 89 (7.0) | 45 (7.3) |  |  | 14 (2.3) | 27 (4.5) | 35 (7.7) |  |
| Asian | 27 (8.9) | 37 (5.3) | 101 (7.9) | 71 (11.5) |  |  | 22 (3.6) | 19 (3.2) | 13 (2.9) |  |
| Other | 25 (8.2) | 38 (5.4) | 71 (5.6) | 45 (7.3) |  |  | 18 (3.0) | 18 (3.0) | 25 (5.5) |  |
| Birth weight (g) |  |  |  |  | <0.001 |  |  |  |  | 0.0076 |
| Low (<2,500) | 47 (16.7) | 57 (9.1) | 247 (21.9) | 129 (22.2) |  |  | 61 (11.1) | 71 (12.7) | 39 (9.0) |  |
| Normal (2,500-4,000) | 213 (75.8) | 507 (80.7) | 825 (73.0) | 425 (73.2) |  |  | 427 (77.8) | 440 (78.4) | 372 (85.5) |  |
| High (>4,000) | 21 (7.5) | 64 (10.2) | 58 (5.1) | 27 (4.7) |  |  | 61 (11.1) | 50 (8.9) | 24 (5.5) |  |
| Extracardiac malformations | |  |  |  | <0.001 |  |  |  |  | 0.042 |
| Yes | 92 (30.3) | 121 (17.1) | 313 (24.4) | 151 (24.4) |  |  | 105 (17.2) | 111 (18.4) | 105 (23.1) |  |
| No | 212 (69.7) | 586 (82.9) | 969 (75.6) | 467 (75.6) |  |  | 506 (82.8) | 491 (81.6) | 349 (76.9) |  |

AVD – aortic valve disease (aortic stenosis, bicuspid aortic valve), COA - coarctation of the aorta, CTD – conotruncal heart defect, DORV - double outlet right ventricle, D-TGA – D-transposition of the great arteries, HLHS – hypoplastic left heart syndrome , LVOT – left ventricular outflow tract, TOF – tetralogy of Fallot, VSD – ventricular septal defect.
^a^ No recognized clinical syndrome but may have noncardiac anomalies.

^b^ Conoventricular, conoseptal hypoplasia and posterior malalignment type ventricular septal defects.

^c^ ANOVA test for continuous variables; chi-square test (or Fisher’s exact test when >20% of cells had an expected cell count <5) for categorical variables.
^d^ Excluded mothers whose highest education was in the United Kingdom.
^e^ Information only available for cases <1 year at recruitment.
^f^ May not sum to total because of missing data.

^g^ Used Welch’s ANOVA because due to heterogeneity of variance.
