# Supplementary material for: The Congenital Heart Disease Genetic Network Study: Cohort description
Source: PLoS One. 2018 Jan 19;13(1):e0191319. doi: 10.1371/journal.pone.0191319 (PMC5774789; doi:10.1371/journal.pone.0191319)
Supplement: S5 Table — AVD—aortic valve disease (aortic stenosis, bicuspid aortic valve), COA—coarctation of the aorta, CTD—conotruncal heart defect, DORV—double outlet right ventricle, D-TGA—D-transposition of the great arteries, HLHS—hypoplastic left heart syndrome, LVOT—left ventricular outflow tract, TOF—tetralogy of Fallot, VSD–ventricular septal defect. (DOCX) [file pone.0191319.s005.docx]

S5 Table. Neurodevelopmental outcomes across major CTD and LVOT subtypes for nonsyndromic^a^ cases >5 years in the Pediatric Cardiac Genetic Consortium Cohort

|  | **CTD** | | | | |  | | **LVOT** | | | |
| --- | --- | --- | --- | --- | --- | --- | --- | --- | --- | --- | --- |
|  | **DORV** | **D-TGA** | **TOF** | **VSD**^b^ | p-value^c^ |  | **AVD** | | **COA** | **HLHS** | p-value^c^ |
| Cases >5 year | n=126 | n=398 | n=706 | n=232 |  |  | n=504 | | n=301 | n=155 |  |
|  | N^e^ (%) | | | |  |  | N^e^ (%) | | | |  |
|  |  |  |  |  |  |  |  |  |  |  |  |
| Attention deficit hyperactivity disorder | |  |  |  | 0.61 |  |  | |  |  | 0.24 |
| Yes | 9 (7.3) | 37 (9.3) | 51 (7.3) | 21 (9.1) |  |  | 51 (10.2) | | 22 (7.4) | 18 (11.9) |  |
| No | 115 (92.7) | 359 (90.7) | 649 (92.7) | 211 (92.0) |  |  | 450 (89.8) | | 276 (92.6) | 133 (88.1) |  |
| Anxiety |  |  |  |  | 0.03 |  |  | |  |  | 0.05 |
| Yes | 7 (5.6) | 53 (13.4) | 61 (8.8) | 21 (9.1) |  |  | 36 (7.2) | | 22 (7.4) | 20 (13.2) |  |
| No | 119 (94.4) | 343 (86.6) | 636 (91.3) | 210 (90.9) |  |  | 464 (92.8) | | 277 (92.6) | 132 (86.8) |  |
| Autism Spectrum |  |  |  |  | 0.36 |  |  | |  |  | 0.74 |
| Yes | 4 (3.2) | 6 (1.5) | 14 (2.0) | 8 (3.5) |  |  | 12 (2.4) | | 5 (1.7) | 4 (2.6) |  |
| No | 122 (96.8) | 390 (98.5) | 688 (98.0) | 224 (96.6) |  |  | 490 (97.6) | | 295 (98.3) | 148 (97.4) |  |
| Behavioral |  |  |  |  | 0.81 |  |  | |  |  | 0.20 |
| Yes | 7 (5.6) | 18 (4.6) | 27 (3.9) | 9 (3.9) |  |  | 14 (2.8) | | 4 (1.3) | 6 (4.0) |  |
| No | 118 (94.4) | 378 (95.5) | 671 (96.1) | 222 (96.1) |  |  | 489 (97.2) | | 296 (98.7) | 144 (96.0) |  |
| Depression |  |  |  |  | 0.46 |  |  | |  |  | 0.60 |
| Yes | 7 (5.6) | 32 (8.1) | 53 (7.6) | 12 (5.2) |  |  | 24 (4.8) | | 10 (3.3) | 6 (3.9) |  |
| No | 119 (94.4) | 363 (91.9) | 644 (92.4) | 220 (94.8) |  |  | 478 (95.2) | | 290 (96.7) | 148 (96.1) |  |
| Developmental Delay |  |  |  |  | 0.33 |  |  | |  |  | <0.001 |
| Yes | 22 (17.5) | 45 (11.4) | 99 (14.1) | 31 (13.5) |  |  | 32 (6.4) | | 27 (9.0) | 46 (30.1) |  |
| No | 104 (82.5) | 350 (88.6) | 603 (85.9) | 199 (86.5) |  |  | 470 (93.6) | | 273 (91.0) | 107 (69.9) |  |
| Learning Disability |  |  |  |  | 0.27 |  |  | |  |  | <0.001 |
| Yes | 26 (20.6) | 83 (21.0) | 125 (17.9) | 35 (15.2) |  |  | 56 (11.2) | | 41 (13.7) | 54 (35.1) |  |
| No | 100 (79.4) | 312 (79.0) | 572 (82.1) | 196 (84.9) |  |  | 446 (88.8) | | 259 (86.3) | 100 (64.9) |  |
| Mental Retardation |  |  |  |  | 0.61 |  |  | |  |  | 0.19 |
| Yes | 4 (3.2) | 7 (1.8) | 14 (2.0) | 3 (1.3) |  |  | 6 (1.2) | | 4 (1.3) | 5 (3.3) |  |
| No | 120 (96.8) | 389 (98.2) | 687 (98.0) | 226 (98.7) |  |  | 495 (98.8) | | 296 (98.7) | 148 (96.7) |  |

AVD – aortic valve disease (aortic stenosis, bicuspid aortic valve), COA - coarctation of the aorta, CTD – conotruncal heart defect, DORV - double outlet right ventricle, D-TGA – D-transposition of the great arteries, HLHS – hypoplastic left heart syndrome, LVOT – left ventricular outflow tract, TOF – tetralogy of Fallot, VSD –ventricular septal defect.

^a^ No recognized clinical syndrome but may have noncardiac anomalies.

^b^ Conoventricular, conoseptal hypoplasia and posterior malalignment type ventricular septal defects.
^c^ Unadjusted logistic regression.
^d^ Composite variable indicating a positive parental report of autism, developmental delay, learning disability, or mental retardation.

^e^ May not sum to total because of missing data.

| S5 Table (cont’d). Neurodevelopmental outcomes across major CTD and LVOT subtypes for nonsyndromic^a^ cases >5 years in the Pediatric Cardiac Genetic Consortium Cohort | | | | | | | | | | | | | |
| --- | --- | --- | --- | --- | --- | --- | --- | --- | --- | --- | --- | --- | --- |
|  | **CTD** | | | | |  | **LVOT** | | | | | | |
|  | **DORV** | **D-TGA** | **TOF** | **VSD**^b^ | p-value^c^ |  | **AVD** | **COA** | | **HLHS** | p-value^c^ | | |
| Cases >5 year | n=126 | n=398 | n=706 | n=232 |  |  | n=504 | n=301 | | n=155 |  |  |  |
|  | N^e^ (%) | | | |  |  | N^e^ (%) | | | |  | | |
| Obsessive-compulsive disorder | |  |  |  | 0.24 |  |  |  | |  | 0.72 | |  |
| Yes | 1 (0.8) | 13 (3.3) | 15 (2.1) | 3 (1.3) |  |  | 9 (1.8) | 4 (1.3) | | 1 (0.7) |  | |  |
| No | 125 (99.2) | 383 (96.7) | 686 (97.9) | 229 (98.7) |  |  | 492 (98.2) | 296 (98.7) | | 153 (99.4) |  | |  |
| Repeated Grade |  | | | | 0.99 |  |  | | | | 0.001 | | |
| Yes | 17 (14.7) | 58 (15.1) | 98 (14.5) | 29 (14.1) |  |  | 42 (8.7) | 18 (6.5) | | 24 (17.4) |  | | |
| No | 99 (85.3) | 325 (84.9) | 580 (85.6) | 177 (85.9) |  |  | 443 (91.3) | 260 (93.5) | | 114 (82.6) |  | | |
| Seizure Disorder |  |  |  |  | 0.47 |  |  |  | |  | 0.001 | | |
| Yes | 4 (3.2) | 16 (4.0) | 22 (3.1) | 4 (1.7) |  |  | 12 (2.4) | 6 (2.0) | | 12 (7.8) |  | | |
| No | 122 (96.8) | 380 (96.0) | 678 (96.9) | 227 (98.3) |  |  | 490 (97.6) | 294 (98.0) | | 141 (92.2) |  | | |
| Speech Problem |  |  |  |  | 0.94 |  |  |  | |  | 0.007 | | |
| Yes | 19 (15.1) | 58 (14.6) | 112 (16.0) | 35 (15.2) |  |  | 64 (12.7) | 37 (12.3) | | 34 (22.2) |  | | |
| No | 107 (84.9) | 339 (85.4) | 589 (84.0) | 196 (84.9) |  |  | 439 (87.3) | 263 (87.7) | | 119 (77.8) |  | | |
| Other |  |  |  |  | 0.11 |  |  |  | |  | 0.94 | | |
| Yes | 2 (1.6) | 14 (3.5) | 10 (1.4) | 3 (1.3) |  |  | 9 (1.8) | 4 (1.3) | | 2 (1.3) |  | | |
| No | 124 (98.4) | 382 (96.5) | 691 (98.6) | 229 (98.7) |  |  | 493 (98.2) | 296 (98.7) | | 150 (98.7) |  | | |
| Composite^d^ |  |  |  |  | 0.24 |  |  |  |  | | | <0.001 | |
| Yes | 33 (26.2) | 105 (26.4) | 172 (24.4) | 45 (19.4) |  |  | 53 (17.6) | 69 (13.7) | 73 (47.1) | | |  | |
| No | 93 (73.8) | 293 (73.6) | 534 (75.6) | 187 (80.6) |  |  | 248 (82.4) | 435 (86.3) | 82 (52.9) | | |  | |

AVD – aortic valve disease (aortic stenosis, bicuspid aortic valve), COA - coarctation of the aorta, CTD – conotruncal heart defect, DORV - double outlet right ventricle, D-TGA – D-transposition of the great arteries, HLHS – hypoplastic left heart syndrome, LVOT – left ventricular outflow tract, TOF – tetralogy of Fallot, VSD –ventricular septal defect.

^a^ No recognized clinical syndrome but may have noncardiac anomalies.

^b^ Conoventricular, conoseptal hypoplasia and posterior malalignment type ventricular septal defects.
^c^ Unadjusted logistic regression.
^d^ Composite variable indicating a positive parental report of autism, developmental delay, learning disability, or mental retardation.

^e^ May not sum to total because of missing data.
